# Effects of Exercise Training and IL-6 Receptor Blockade on Gastric Emptying and GLP-1 Secretion in Obese Humans: Secondary Analyses From a Double Blind Randomized Clinical Trial

**DOI:** 10.3389/fphys.2019.01249

**Published:** 2019-10-04

**Authors:** Louise Lang Lehrskov, Regitse Højgaard Christensen, Anne-Sophie Wedell-Neergaard, Grit Elster Legaard, Emma Dorph, Monica Korsager Larsen, Marie Henneberg, Natja Launbo, Sabrina Ravn Fagerlind, Sidsel Kofoed Seide, Stine Nymand, Maria Ball, Nicole Vinum, Camilla Dahl, Nicolai Jacob Wewer Albrechtsen, Jens Juul Holst, Mathias Ried-Larsen, Jaya Birgitte Rosenmeier, Rikke Krogh-Madsen, Kristian Karstoft, Bente Klarlund Pedersen, Helga Ellingsgaard

**Affiliations:** ^1^Centre of Inflammation and Metabolism, Centre for Physical Activity Research, Rigshospitalet, University of Copenhagen, Copenhagen, Denmark; ^2^Department of Biomedical Sciences, NNF Center for Basic Metabolic Research, Faculty of Health and Medical Sciences, University of Copenhagen, Copenhagen, Denmark; ^3^Department of Clinical Biochemistry, Rigshospitalet, University of Copenhagen, Copenhagen, Denmark; ^4^Department of Cardiology, Bispebjerg Hospital, Capital Region of Denmark, University of Copenhagen, Copenhagen, Denmark

**Keywords:** interleukin-6, gastric emptying, exercise, tocilizumab, glucagon, GLP-1

## Abstract

**Background:**

Interleukin-6 (IL-6) is released from skeletal muscle during exercise and systemic IL-6 levels therefore increase acutely in response to a single bout of exercise. We recently showed that an acute increase in IL-6 delayed gastric emptying rate and improved postprandial glycemia. Here we investigate whether repeated increases in IL-6, induced by exercise training, influence gastric emptying rate and moreover if IL-6 is required for exercise-induced adaptations in glycemic control including secretion of glucagon and glucagon-like peptide-1 (GLP-1).

**Methods:**

A total of 52 abdominally obese non-diabetic men and women were randomly assigned into four groups performing 12 weeks of endurance exercise or no exercise with or without IL-6 receptor blockade (tocilizumab). The primary endpoint was change in gastric emptying rate in response to the intervention and other endpoints included changes in glycemic control, glucagon, and GLP-1 secretion.

**Results:**

There was no change in gastric emptying rate in any of the four groups following the intervention and comparing differences in change between groups also revealed no difference. Postprandial glucose remained unchanged in all groups but the exercise + tocilizumab group, which improved postprandial glucose in response to the intervention. The area under the curve for meal-stimulated glucagon, active and total GLP-1 increased in response to IL-6 receptor blockade, this effect was independent of exercise.

**Conclusion:**

Exercise training and long-term IL-6 receptor blockade did not change gastric emptying rates in obese humans. IL-6 receptor blockade increased glucagon and GLP-1 secretion and implicate IL-6 in the regulation of the human alpha and L cells.

## Introduction

Gastric emptying is a critical regulator of postprandial glucose contributing to as much as 35% of peak glucose following a meal (Horowitz et al., 1993). Postprandial glucose levels are important for glycated hemoglobin (HbA1c) (Ceriello et al., 2004) emphasizing the relevance of in-depth understanding of the physiological regulators of gastric emptying.

Interleukin-6 (IL-6) is a cytokine influencing glucose homeostasis (Matthews et al., 2010). We recently showed that acutely increased IL-6, following an IL-6 infusion, delayed gastric emptying rate and improved postprandial glucose (Lehrskov et al., 2018). Consistent with these effects, IL-6 receptor blockade enhanced the rate of gastric emptying after an acute bout of exercise suggesting that IL-6 regulation of gastric emptying may be a physiological phenomenon (Lehrskov et al., 2018).

IL-6 is also a myokine released from skeletal muscle in response to exercise (Pedersen et al., 2001). During exercise, systemic IL-6 levels increase acutely and return to baseline within hours (Fischer, 2006). Exercise on a regular basis (training) is therefore associated with repeated increases in plasma IL-6. Whether exercise-induced IL-6 plays a role in the metabolic adaptations to exercise training is unclear. In some ways, IL-6 regulation of glucose metabolism bears resemblances to the effects of exercise on glucose metabolism. Infusion of IL-6 has shown beneficial effects of IL-6 on peripheral glucose uptake (Febbraio et al., 2004; Carey et al., 2006) and insulin stimulated glucose uptake (Carey et al., 2006). Whether repetitive increases in IL-6 induced by exercise training changes gastric emptying rate has not been investigated.

Most studies investigating exercise effects on gastric emptying have studied the acute effect of a single bout of exercise on gastric emptying during a post exercise meal (Horner et al., 2015b). A meta-analysis of these studies revealed a possible effect of exercise intensity on gastric emptying (Horner et al., 2015b). The mechanism behind this exercise intensity-dependent regulation of gastric emptying has not been identified.

The incretin hormone, glucagon-like peptide-1 (GLP-1) is secreted from the intestinal L cells in response to nutrients; however, GLP-1 has also been shown to increase during an acute exercise bout (Martins et al., 2007) and some studies have demonstrated an increased secretory capacity of the L cell following an exercise training intervention (Chanoine et al., 2008; Martins et al., 2010).

IL-6 stimulates GLP-1 secretion from L cells under certain circumstances *in vitro* (Ellingsgaard et al., 2011); and in rodents, exercise-induced GLP-1 was found to be dependent on IL-6 (Ellingsgaard et al., 2011). A link between training-induced adaptations of the L cell and IL-6 signaling has not been described in humans. The pancreatic alpha cell has been shown to be under the influence of IL-6, and *in vitro* and *in vivo* studies have shown that IL-6 stimulates glucagon secretion (Ellingsgaard et al., 2008; Tweedell et al., 2011; Fernández-Millán et al., 2013; Chow et al., 2014; Lehrskov et al., 2018). Whether long-term IL-6 receptor blockade influences glucagon secretion has not been demonstrated.

To investigate the role of IL-6 in training-induced adaptations of gastric emptying, glycemic control, and GLP-1 secretion, abdominally obese inactive individuals performed a 12-week exercise training intervention or no exercise training (control) in the presence and absence of the IL-6 receptor blockade (tocilizumab). We hypothesized that repetitive increases in IL-6 induced by exercise training would decelerate gastric emptying rate, and moreover that this effect would be abolished in the presence of IL-6 receptor blockade.

## Materials and Methods

### Study and Participants

This study was a randomized exercise training intervention study with the overall primary endpoint to investigate the role of IL-6 in exercise training-induced regulation of visceral adipose tissue mass (Wedell-Neergaard et al., 2018). Data regarding visceral adipose tissue are published previously (Wedell-Neergaard et al., 2018). The study was performed between August 2016 and April 2018 at the Centre for Physical Activity Research (CFAS) in Copenhagen, Denmark. The study was approved by the ethical committee of the Capital Region of Denmark (H-16018062) and reported to the Danish Data Protection Agency (2012-58-0004). The study was registered at ClinicalTrials.gov NCT02901496 and was conducted in accordance with the guidelines for Good Clinical Practice and the Declaration of Helsinki. All participants provided written informed consent.

The full study protocol is published (Christensen et al., 2018). Briefly, participants were eligible if they were physically inactive (defined as less than 2.5 h of physical activity per week) (Tremblay et al., 2017) and abdominally obese (waist to height ratio ≥ 0.5 and/or waist circumference ≥ 88/102 cm for women/men, respectively) and age > 18 years old. Key exclusion criteria were diabetes (HbA1c ≥ 48 mmol/mol or fasting glucose ≥ 7.0 mmol/l), pregnancy or breastfeeding, ischemic heart disease, infectious or immunosuppressive disease, treatment with NSAID, treatment with biologic drugs for rheumatic diseases, systemic prednisolone, other immunotherapy or health conditions that prevented participation in the exercise intervention (e.g., severe obesity). Permitted medication should be taken at a stable dose for at least 4 weeks prior to randomization and preferably remain stable throughout the study period.

Data related to adverse events are published (Wedell-Neergaard et al., 2018).

### Randomization

**FIGURE 1:**
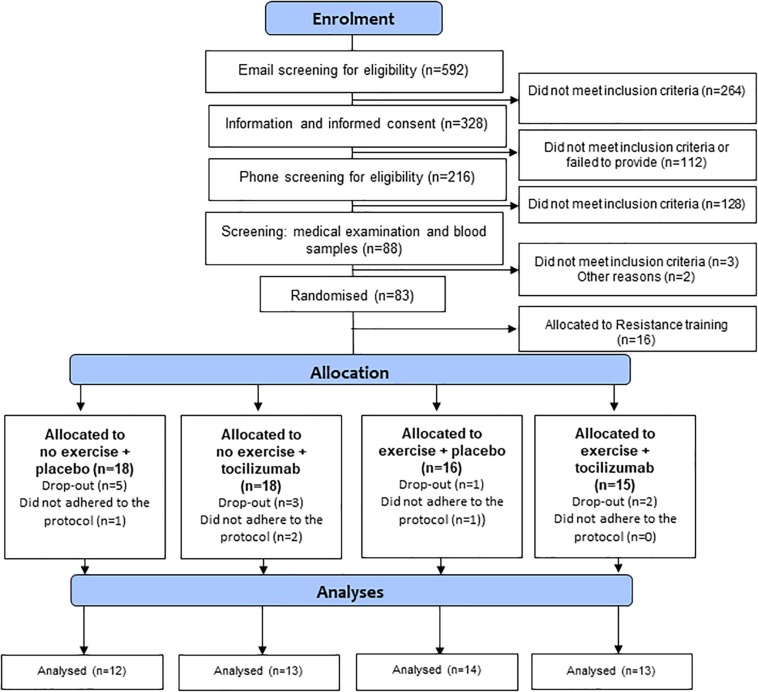
Flowchart.

Eligible participants were block-randomized (1:1:1:1:1) into five groups as described in the protocol paper (Christensen et al., 2018). The randomization sequence was generated by one researcher who did not participate in any clinical study procedures (KK) and concealed from all other researchers. The principal study investigators thus remained blinded to the training modality as well as the infusions (tocilizumab/saline). The five randomized groups were: no exercise + placebo, no exercise + tocilizumab, exercise + placebo, exercise + tocilizumab, or resistance training + placebo as described in the protocol paper (Christensen et al., 2018), all participants except those allocated to resistance training were included in the analyzes of this particular study (Figure 1).

### IL-6 Receptor Blockade (Tocilizumab)

The recombinant humanized monoclonal IL-6 receptor antibody tocilizumab (Roche, Basel, Switzerland) was reconstituted in saline and a total volume of 100 ml was infused monthly over 1 h at the maximal recommended dose of 8 mg/ml or a maximum of 800 mg (RoActemra | European Medicines Agency, n.d.). European Public Assessment Report for RoActemra. The concentration of tocilizumab following infusion of 8 mg/kg tocilizumab ranges from 10 to 183 μg/ml. Complete blockade is achieved at 4 μg/ml (Song and Yoshizaki, 2015). Given the half-life of tocilizumab of 13 days, it can be anticipated tocilizumab fully blocked IL-6 actions during the intervention period.

### Study Visits and Interventions

Before and after the intervention all participants went through several study visits including medical screening, blood sampling, mixed meal tolerance tests (MMTT), Dual X-ray Absorptiometry (DXA) scans, and maximal oxygen consumption rate (VO_2_ max) tests. Continuous glucose monitoring (CGM) and diet registration were performed over a 3-day period before and after the intervention. The intervention consisted of 12 weeks of supervised endurance exercise or no exercise combined with monthly infusion of tocilizumab. Saline was infused as placebo. Each exercise bout was 45 min high-intensity interval training and was performed on an ergometer bicycle. The intensity progressively increased over the 12-week training period. Safety and compliance were assessed every 4 weeks.

### DXA and VO_2_ Max

Dual X-ray Absorptiometry scans were used to assess changes in body composition (Lunar Prodigy Advance, GE Medical systems, Milwaukee, United States). VO_2_ max was determined during a bicycle ergometer test (Monark Ltd., Varberg, Sweden). After a 5 min warm-up at 70 w, the load was increased by 15 w every minute until exhaustion. Ventilation rate, oxygen uptake and expired carbon dioxide levels as well as heart rate were measured throughout the duration of the test (Quark b2, Cosmed, Rome, Italy). A test was valid if the respiratory exchange ratio > 1.1, a plateau in oxygen intake was reached or the heart rate was ±10 heartbeats from the estimated maximal heart rate (Edvardsen et al., 2014). The test result was used to determine the exercise workload for participants randomized to exercise and to assess changes in aerobic capacity in all four groups following the intervention.

### CGM, Free-Living Physical Activity, and Diet Registration

To obtain insight into everyday glycemic control, a continuous glucose sensor (Enlite, Medtronic, Friedly, MN, United States) was inserted into the abdominal subcutaneous adipose tissue of all participants. The sensor was connected to a CGM monitor (iPro2, Medtronic) and calibrated four times daily with a glucometer (Contour, Bayer, Basel, Switzerland). The obtained data were analyzed for mean amplitude glycemic excursions (MAGE) a marker for glycemic variability (Service et al., 1970). Self-reported dietary intake was registered over 3 days before and after the intervention. Average daily calorie intake was calculated using madlog-vita.dk. Free-living physical activity was assessed using accelerometry (AX3, Newcastle, United Kingdom), sampling frequency 100 Hz, over 4 days before and after the intervention (Treuth et al., 2004).

### Mixed Meal Tolerance Test

The mixed meal was a 360 mL liquid meal containing 385 kcal. The energy distribution was 15% fat, 20% protein, and 65% carbohydrate. The meal contained 1.5 g paracetamol to allow assessment of gastric emptying rate (Heading et al., 1973). The meal was ingested over less than 2 min. To measure paracetamol concentrations and parameters of glycemic control blood samples were drawn regularly (−10, 0, 15, 30, 60, 90, 120, 150, and 180 min) relative to ingestion of the meal. To ensure that no acute effects of exercise impacted on the MMTT, exercise was not allowed 48 h prior to the test.

### Blood Samples and Analysis

Baseline blood parameters, safety blood parameters, HbA1c, glucose, insulin, and paracetamol were measured at the Department of Clinical Biochemistry, Rigshospitalet, Copenhagen University Hospital. Plasma levels of total GLP-1 were determined using a radioimmunoassay developed in-house (codename 390) that is COOH-terminal and therefore measuring GLP-1 (7-36NH2 as well as the metabolite GLP-1 9-36NH2) as described previously (Orskov et al., 1994). Plasma levels of active GLP-1 was assessed with a commercially assay (Alpco, Salem, NH, United States). Plasma glucagon were measured using a C-terminal based radioimmunoassay (codename 4305) as described previously (Wewer Albrechtsen et al., 2014). Inter and intra assay variations for the GLP-1 and glucagon assays were less than 15 and 6%, respectively, and the lower limit of quantification was around 1 pmol/L with a precision of 1 pmol/L (Wewer Albrechtsen et al., 2015).

### Modeling Insulin Sensitivity, Secretion, and Disposition Index

Whole body insulin sensitivity index was calculated by the Matsuda and DeFronzo method as follows (Matsuda and DeFronzo, 1999).

Whole body insulin sensitivity index

$$=10000-\sqrt{\left(\mathrm{mean}\,\mathrm{insulin}\times \mathrm{mean}\,\mathrm{glucose}\,\mathrm{during}\,\mathrm{MMTT}\right)}$$

×(fasting⁢glucose×fasting⁢insulin)

The insulinogenic index was calculated based on insulin and glucose levels after the meal load during the MMTT (Phillips et al., 1994; Wareham et al., 1995):

(Insulin 30min-fastinginsulin)/

  ⁢(glucose⁢ 30⁢min-fasting⁢glucose)

The oral disposition index was assessed during the first 30 min of the MMTT as Matsuda × the insulinogenic index (Solomon et al., 2014).

### Study Endpoints

The main outcome was change in gastric emptying rate determined as changes in paracetamol kinetics following the intervention. Other outcomes included changes in postprandial plasma levels of glucose, insulin. Moreover, changes in postprandial plasma levels of glucagon, total and active GLP-1, as well as changes in whole body insulin sensitivity index, insulinogenic index, and the oral disposition index, were assessed as explorative outcomes. Also changes in HbA1c and glycemic variability were considered as explorative outcomes.

### Statistical Analysis

Results are expressed as mean ± SEM in figures and as mean ± SD in Table 1. Changes within groups and differences in change between groups are expressed as mean (95% CI).

**TABLE 1:** Participant characteristics and intervention-induced changes within groups.

	**No exercise + placebo**		**No exercise + tocilizumab**		**Exercise + placebo**		**Exercise + tocilizumab**	
	**Before**	**After**	**Before**	**After**	**Before**	**After**	**Before**	**After**
*n* (male/female)	12 (1/11)		13 (5/8)		14 (3/11)		13 (3/10)	
Age (years)	48 (12)		44 (12)		39 (13)		44 (12)	
**Body composition**								
Body mass (kg)	95.4 (19.8)	96.0 (19.7)	98.8 (16.9)	99.5 (16.9)	92.4 (13.5)	91.8 (13.6)	95.5 (14.5)	96.4 (15.3)
**Lipids (mmol/l)**								
Total cholesterol	5.1 (0.8)	5.2 (1.0)	5.1 (1.0)	5.6 (1.0)	4.8 (0.9)	4.7 (0.9)	4.8 (0.7)	5.2^∗^(0.6)
HDL-cholesterol	1.3 (0.3)	1.3 (0.3)	1.3 (0.3)	1.3 (0.4)	1.4 (0.3)	1.4 (0.3)	1.4 (0.3)	1.5 (0.4)
LDL-cholesterol	3.3 (0.8)	3.4 (0.7)	3.3 (0.9)	3.6^∗^(0.9)	3.1 (0.6)	3.0 (0.7)	3.1 (0.7)	3.3 (0.5)
**Blood pressure (mmHg)**								
Systolic	123 (20)	124 (7)	126 (18)	128 (16)	128 (21)	127 (14)	134 (20)	133 (18)
Diastolic	84 (9)	85 (6)	86 (13)	82 (16)	87 (10)	83 (10)	89 (9)	85 (10)
**Glycemic control**								
HbA1c (%)	6.2 (0.6)	6.2 (0.4)	6.3 (0.4)	6.2 (0.4)	5.8 (0.4)	5.9^∗^(0.4)	5.8 (0.4)	5.8 (0.4)
HbA1c (mmol/mol)	36.5 (3.8)	36.7 (3.0)	37.2 (2.7)	36.8 (2.9)	33.6 (3.0)	35.0^∗^(2.6)	34.0 (3.0)	34.0 (2.6)
Fasting glucose (mmol/l)	5.0 (0.5)	5.1 (0.5)	5.3 (0.9)	5.4 (0.8)	5.1 (0.5)	5.3 (0.7)	5.0 (0.5)	5.0 (0.4)
Fasting insulin (pmol/l)	100 (40)	116 (82)	92 (58)	96 (51)	111 (78)	94 (52)	88 (55)	92 (56)
Glycemic variability, MAGE (CGM)	2.1 (1.1)	1.8 (0.7)	1.9 (0.4)	1.6 (0.5)	1.8 (0.6)	1.4 (0.6)	1.5 (0.4)	1.4 (0.3)
**Postprandial glycemic control**								
Matsuda (MMTT)	3.0 (1.0)	3.3 (1.4)	4.7 (2.8)	4.6 (2.9)	3.4 (1.4)	3.9 (1.9)	3.8 (2.5)	4.5 (2.7)
Insulin secretion index (MMTT)	353 (282)	474 (400)	333 (178)	522 (738)	431 (191)	390 (126)	485 (483)	574 (365)
Oral disposition index (MMTT)	1031 (959)	1498 (1429)	1232 (632)	1846 (2184)	1502 (1040)	1477 (719)	1453 (922)	2093^∗^(865)

*Data are presented as mean (SD) except from sex. ^∗^*p* < 0.05 determined by Student’s paired *t*-test comparing intervention-induced changes within groups.*

The level of significance was set at *p* < 0.05. Statistical analyses were performed using GraphPad Prism version 7.02 (GraphPad Software, La Jolla, CA, United States). Within-group differences in plasma concentrations of paracetamol, glucose, insulin, glucagon, total GLP-1, and active GLP-1 were determined using the two-way repeated-measure analysis of variance (RM-ANOVA). Within-group differences in AUC were analyzed using Student’s paired *t*-test and differences in change between groups were assessed using the one-way ANOVA and Student’s unpaired *t*-test.

## Results

### Study Participants

We enrolled 56 participants in the study. Due to protocol violations four participants were excluded from the data analysis. The remaining 52 participants were randomized to no exercise + placebo (*n* = 12), no exercise + tocilizumab (*n* = 13), exercise + placebo (*n* = 14), and exercise + tocilizumab (*n* = 13) and included in the per protocol analysis (Figure 1). In the CGM analysis another five participants were excluded due to incomplete availability of data. Data from the remaining 47 participants, distributed as follows: no exercise + placebo (*n* = 11), no exercise + tocilizumab (*n* = 12), exercise + placebo (*n* = 11), and exercise + tocilizumab (*n* = 13) were included in the CGM analysis. Participant characteristics and intervention-induced changes are shown in Table 1.

Data in Table 1 are published (Wedell-Neergaard et al., 2018) except for the changes in HbA1c, glycemic variability and parameters of postprandial glycemic control.

### VO_2_ Max, Training Compliance, Body Weight, Free-Living Physical Activity, and Dietary Intake

As anticipated, VO_2_ max increased in the exercise + placebo group and in the exercise + tocilizumab group. All participants in the two training groups complied with the minimum of 29 training sessions, and compliance was similar in the two groups. Body weight remained unchanged in all groups following the intervention (Table 1), and there were no differences in free-living physical activity and dietary intake between the groups. All data described in this section can be found in our previously published paper (Wedell-Neergaard et al., 2018).

### Circulating Levels of IL-6

As expected, baseline plasma levels of IL-6 increased in response to tocilizumab (Zhang et al., 2017). Exercise training for 12 weeks had no effect on baseline IL-6 levels. These data are previously reported (Wedell-Neergaard et al., 2018). IL-6 increased significantly in response to an acute exercise bout in the exercise + placebo group from 0.77 pg/ml (SD: 0.27) to 1.14 pg/ml (SD: 0.39) and from 10.23 pg/ml (SD: 5.25) to 11.33 pg/ml (SD: 5.18) in the exercise + tocilizumab group.

### Gastric Emptying

**FIGURE 2:**
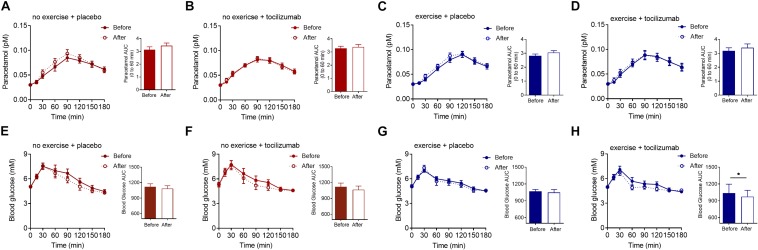
Paracetamol and glucose. **(A–D)** Plasma paracetamol concentrations during a MMTT (left) and paracetamol AUC 0–60 min (right) before and after **(A)** no exercise + placebo, **(B)** no exercise + tocilizumab, **(C)** exercise + placebo, and **(D)** exercise + tocilizumab. **(E–H)** Plasma glucose concentrations during a MMTT (left) and blood glucose AUC before and after **(E)** no exercise + placebo, **(F)** no exercise + tocilizumab, **(G)** exercise + placebo, and **(H)** exercise + tocilizumab. Tocilizumab is an IL-6 receptor antibody. Data represent mean ± SEM. ^∗^*p* < 0.05 using Student’s paired *t*-test (right).

In contrast to our hypothesis there was no effect of exercise training, IL-6 receptor blockade, or the combination of the two, on the appearance of paracetamol into the circulation (Figure 2). Time to appearance, rate of appearance (Figure 2) and area under the curve (0–60 min) (Figure 2 and Table 2) remained unchanged in all four groups following the intervention and there was no difference in change between groups (Table 2). Hence, exercise training and long-term IL-6 receptor blockade did not affect gastric emptying in the present study.

**TABLE 2:** Intervention-induced changes during a mixed meal tolerance test within and between groups.

	**No exercise + placebo**	**No exercise + tocilizumab**	**Exercise + placebo**	**Exercise + tocilizumab**	**No exercise + placebo vs. No exercise + tocilizumab**	**No exercise + placebo vs. Exercise + placebo**	**No exercise + placebo vs. Exercise + Tocilizumab**	**Exercise + placebo vs. Exercise + Tocilizumab**
Paracetamol (0–60 min)	0.3 (−0.2 to 0.8)	0.1 (−0.2 to 0.4)	0.2 (−0.1 to 0.6)	0.2 (−0.2 to 0.7)	−0.2 (−0.8 to 0.4)	0.1 (−0.5 to 0.7)	0.1 (−0.6 to 0.7)	0.0 (−0.5 to 0.5)
Blood glucose	−36.0 (−136.0 to 64.1)	−55.8 (−132.7 to21.1)	−19.3 (−84.6 to 46.0)	−62.3 (−124.0 to −0.6)	−19.8 (−137.8 to 98.2)	16.8 (−931 to 126.6)	−26.3 (−135.2 to 82.7)	−43.0 (−128.7 to 42.6)
Insulin	5443 (−14128 to 25013)	−1908 (−16195 to 12380)	−7166 (−20577 to 6242)	−10637 (−26567 to 5293)	−7351 (−29960 to 15259)	12608 (−9285 to 34502)	−16080 (−39741 to 7581)	−3471 (−23130 to 16187)
Glucagon	112.5 (−70.0 to 295.0)	168.8 (17.4 to 320.3)	61.8 (−113.5 to 237.0)	211.8 (28.8 to 394.9)	56.4 (−164.8 to 277.6)	−50.7 (−292.4 to 191.1)	99.4 (−146.2 to 345.0)	150.1 (−90.5 to 390.6)
Total GLP-1	78.2 (−355.5 to 511.9)	333.5 (73.1 to 594.0)	−28 (−308.2 to 252.2)	349 (34.1 to 664.0)	255.4 (−202.8 to 713.5)	−106.2 (−573.5 to 361.1)	270 (−22.8 to 764.5)	377 (21.9 to 775.9)
Active GLP-1	32.0 (−99.5 to 163.4)	448.0 (49.4 to 846.7)	113.8 (−213.9 to 441.4)	412.1 (−23.88 to 848.2)	416.1^∗^ (20.5 to 811.6)	81.8 (−275.8 to 493.4)	380.2 (−48.9 to 809.3)	298.4 (−208.8 to 805.6)
Matsuda (MMTT)	0.3 (−0.5 to 1.0)	−0.1 (−1.4 to 1.1)	0.6 (−0.2 to 1.3)	0.8 (−0.3 to 2.0)	−0.4 (−1.7 to 0.9)	−0.3 (−1.3 to 0.7)	0.6 (−0.7 to 1.9)	0.3 (−1.0 to 1.5)
Insulin secretion index (MMTT)	121.2 (−33.0 to 275.3)	189.5 (−233.7 to 612.6)	−40.8 (−151.4 to 69.7)	89.4 (−50.8 to 229.5)	68.3 (−356.0 to 492.7)	162.0 (−13.8 to 337.7)	−31.8 (−228.1 to 164.5)	130.2 (−36.5 to 296.9)
Oral disposition index (MMTT)	467.0 (−304.6 to 1239)	631.7 (−653.2 to 1881)	−24.8 (−648.4 to 598.8)	640.4 (120.1 to 1161)	146.7 (−1251 to 1545)	491.8 (−436.7 to 1420)	173.4 (−703.5 to 1050)	665.3 (−121.6 to 1452)

*Data are presented as mean change (95% CI) in AUC. ^∗^*p* < 0.05 determined by one-way ANOVA and Student’s unpaired *t*-test comparing differences in change between groups. Tocilizumab is an IL-6 receptor antibody.*

### Glycemic Control

Postprandial glucose remained generally unaffected by training and IL-6 receptor blockade (Figure 2 and Table 2), however, in the exercise + tocilizumab group glucose AUC was significantly reduced following the intervention (Figure 2). Comparing differences in change in glucose AUC between groups showed no difference (Table 2).

The average glucose concentration, assessed by HbA1c, revealed only small and mostly non-significant changes following the intervention (Table 1). Consistent with the overall subtle changes in glycemic control, assessment of glycemic variability, determined as the MAGE, did not change in response to any of the interventions (Table 1). Fasting plasma glucose levels were within the normal range and less than 5.4 mM at baseline and remained unchanged in all groups following the intervention (Table 1).

### Insulin, Glucagon, and GLP-1

**FIGURE 3:**
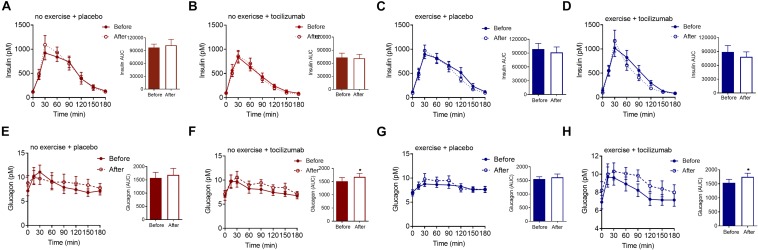
Insulin and glucagon. **(A–D)** Plasma insulin concentrations during a mixed meal tolerance tests (MMTT) (left) and insulin AUC (right) before and after **(A)** no exercise + placebo, **(B)** no exercise + tocilizumab, **(C)** exercise + placebo, and **(D)** exercise + tocilizumab. **(E–H)** Plasma glucagon concentrations during a MMTT (left) and glucagon AUC (right) before and after **(E)** no exercise + placebo, **(F)** no exercise + tocilizumab, **(G)** exercise + placebo, and **(H)** exercise + tocilizumab. Tocilizumab is an IL-6 receptor antibody. Data represent mean ± SEM. ^∗^*p* < 0.05 using Student’s paired *t*-test (right).

To further elaborate on the adaptations related to glycemic control we assessed changes in plasma insulin, glucagon and GLP-1. Plasma levels of fasting and meal-stimulated insulin remained unchanged following the intervention (Tables 1, 2, and Figure 3). Modeling insulin sensitivity and insulin secretion using the Matsuda (Matsuda and DeFronzo, 1999) and insulinogenic index (Phillips et al., 1994; Wareham et al., 1995) also revealed no effect of the interventions (Table 1). In line with reduced postprandial glucose in the exercise + tocilizumab group, the oral disposition index increased in this group following the intervention (Table 1), however, comparing the changes in disposition index between groups showed no significant difference in change (Table 2).

Despite vague changes in glycemia and no changes in insulin secretion, a significant stimulatory effect of IL-6 receptor blockade on meal-induced glucagon secretion (AUC) was observed in both groups receiving tocilizumab (Figure 3). Comparing the changes in glucagon secretion between groups revealed no significant difference in change (Table 2).

**FIGURE 4:**
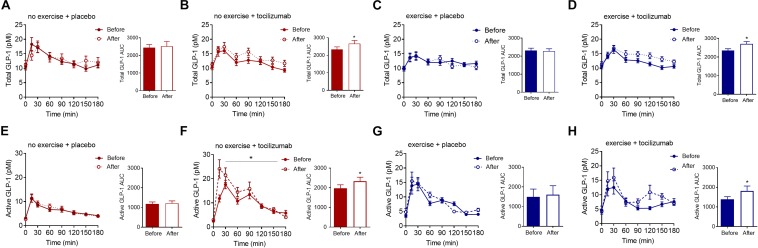
Total and active GLP-1. **(A–D)** Plasma concentrations of total GLP-1 during a MMTT (left) and total GLP-1 AUC (right) before and after **(A)** no exercise + placebo, **(B)** no exercise + tocilizumab, **(C)** exercise + placebo, and **(D)** exercise + tocilizumab. **(E–H)** Plasma concentrations of active GLP-1 during a MMTT (left) and active GLP-1 AUC (right) before and after **(E)** no exercise + placebo, **(F)** no exercise + tocilizumab, **(G)** exercise + placebo, and **(H)** exercise + tocilizumab. Tocilizumab is an IL-6 receptor antibody. Data represent mean ± SEM. ^∗^*p* < 0.05 using Student’s paired *t*-test (right).

The AUC for total and active GLP-1 increased in response to tocilizumab independently of exercise (Figure 4). Comparing changes in active and total GLP-1 between groups mostly revealed no significant difference in change (Table 2).

## Discussion

Given that we previously observed a delay in gastric emptying following an acute increase in IL-6 (Lehrskov et al., 2018) we hypothesized that repetitive increases of IL-6, induced by exercise training, would also delay gastric emptying, but in contrast to our hypothesis exercise training had no effect on gastric emptying in this study. Our knowledge about the long-term effects of exercise training on gastric emptying is primarily based on cross-sectional data which suggests increased gastric emptying rate in trained individuals (Horner et al., 2011, 2015a). Studies investigating the effect of a single bout of exercise on gastric emptying during an immediate post exercise meal have demonstrated a role for exercise intensity, where increased exercise intensity is related to increased gastric emptying rate (Horner et al., 2015b). Nevertheless, our data showed no effect of a 12-week exercise-training period on gastric emptying in physically inactive and abdominally obese people. Whether the absence of an effect is related to exercise intensity or merely reflects that exercise training does not change gastric emptying remains unclear.

Gastric emptying also remained unchanged following long-term IL-6 receptor blockade. We have however, previously observed that the same dose of IL-6 receptor blockade increased gastric emptying during a post exercise meal (Lehrskov et al., 2018) suggesting that exercise-induced IL-6 delays gastric emptying. In the present study gastric emptying was assessed when IL-6 levels were at baseline and it is possible that tocilizumab regulates gastric emptying only when IL-6 levels are elevated, such as following an exercise bout. Given the pharmacokinetic profile of tocilizumab, we can anticipate that the IL-6 receptor blockade was complete during the post intervention assessments (Song and Yoshizaki, 2015). Increased plasma levels of IL-6 and reduced CRP levels support this. Training-induced adaptations of glycemic control were surprisingly mild, however, given that participants were normoglycemic at baseline prior to the training intervention the shortfall of improvements may be a reflection of a poor potential for change. One of the early adaptations to training is improved insulin sensitivity (Dela et al., 1992; Kirwan et al., 2009), yet modeling insulin sensitivity using the Matsuda index revealed no effect of training alone. Combining exercise training and IL-6 receptor blockade significantly reduced postprandial glucose excursions, which combined with no change in insulin resulted in a tendency toward improved Matsuda index and improved disposition index. Such an effect of long-term IL-6 blockade is in contrast to the insulin sensitizing effect observed in response to an infusion of IL-6 (Carey et al., 2006) and suggest that the improved insulin sensitivity may be secondary to IL-6 blockade.

That IL-6 receptor blockade increased glucagon secretion is quite contradictory to studies in rodent and human alpha cells (Ellingsgaard et al., 2008; Fernández-Millán et al., 2013) and to *in vivo* studies in rodents (Tweedell et al., 2011; Chow et al., 2014) and humans (Lehrskov et al., 2018) which all have demonstrated increased glucagon secretion in response to IL-6. However, while most of these studies evaluated the acute effect of increased IL-6, the present study investigated the consequence of long-term IL-6 receptor blockade. Thus, the stimulatory effect of tocilizumab on glucagon secretion may not reflect the inhibition of a direct effect of IL-6 on the alpha cell, but rather secondary adaptations of IL-6 receptor blockade. Participants receiving tocilizumab increased visceral adipose tissue mass (Wedell-Neergaard et al., 2018) and showed similar trends for whole body fat mass; therefore on a very speculative note, the augmented glucagon secretion could be secondary to steatosis (Suppli et al., 2016).

That both active and total GLP-1 levels were elevated in response to tocilizumab suggests an effect on the secretion of GLP-1 (Kuhre et al., 2015) rather than on a DPP-4 mediated modulation of GLP-1. Increased GLP-1 secretion in response to IL-6 receptor blockade may seem surprising given the stimulatory effect of IL-6 on GLP-1 secretion in GLUTag cells (Ellingsgaard et al., 2011). In humans, however, infusion of IL-6 had no effect on GLP-1 secretion, whereas infusion of IL-6 receptor blockade increased GLP-1 (Lehrskov et al., 2018). Thus, it seems that in humans both acute and long-term IL-6 receptor blockade increases meal-stimulated GLP-1 secretion. As for glucagon, it is unclear whether the effect of IL-6 receptor blockade on GLP-1 secretion is a consequence of inhibiting direct effects of IL-6 on L cells or a result of secondary adaptations to long term IL-6 blockade. Increased gastric emptying leads to increased GLP-1 secretion (Holst et al., 2016) which may explain the increased GLP-1 secretion following acute IL-6 receptor blockade (Lehrskov et al., 2018), long-term use of IL-6 receptor blockade did, however, not affect gastric emptying, suggesting that a different mechanism is involved in the regulation of GLP-1 secretion following long-term IL-6 receptor blockade.

Based on the available data there are no clear implications of the increased postprandial glucagon and GLP-1 secretion. Small numerical improvements in postprandial glucose were observed (significant in the exercise + tocilizumab group), but these were not associated with changes in insulin secretion or insulin sensitivity. Of note, both glucagon and GLP-1 levels remained unchanged in the fasting condition and only increased in response to the mixed meal, and whether these meal-associated changes have implications for long-term glycemic control remains unclear.

In conclusion, our study demonstrated that in obese and physically inactive people, gastric emptying rates are unaffected by exercise training and IL-6 receptor blockade. The study also showed that IL-6 receptor blockade increased glucagon and GLP-1 secretion and thus implicates IL-6 in the regulation of the human alpha and L cells.

### Limitations

Assessing gastric emptying using the paracetamol approach may be a limitation as it may not be a sensitive enough method to detect training-induced changes. Despite that participants were abdominally obese and physically inactive they were normoglycemic at baseline, this should be taken into account when evaluating the data on glycemic control. The power analysis of the study was based on changes in visceral adipose tissue mass (Christensen et al., 2018; Wedell-Neergaard et al., 2018) and not on changes in gastric emptying and glycemic control, it is therefore possibly that the secondary analyses were underpowered. Moreover, the sample size was relatively small and conclusions should be interpreted with caution.

## Data Availability Statement

The datasets generated for this study are available on request to the corresponding author.

## Ethics Statement

The study was approved by the ethical committee of the Capital Region of Denmark (H-16018062) and reported to the Danish Data Protection Agency (2012-58-0004).

## Author Contributions

BP, HE, RK-M, KK, JR, MR-L, A-SW-N, RC, and LL conceived and designed the study. LL, RC, A-SW-N, GL, ED, ML, MH, NL, SF, SS, SN, MB, NV, and CD performed the experiments. LL, HE, NW, and JH analyzed and interpreted the data. BP primarily provided the resources. LL and HE wrote the original draft and visualized the data. All authors contributed to the writing, reviewing, and editing of the manuscript.

## Conflict of Interest

The authors declare that the research was conducted in the absence of any commercial or financial relationships that could be construed as a potential conflict of interest.

## References

[B1] Carey A. L. Steinberg G. R. Macaulay S. L. Thomas W. G. Holmes A. G. Ramm G. (2006). Interleukin-6 increases insulin-stimulated glucose disposal in humans and glucose uptake and fatty acid oxidation in vitro via AMP-activated protein kinase. *Diabetes Metab. Res. Rev.* 55 2688–2697. 17003332 10.2337/db05-1404

[B2] Ceriello A. Hanefeld M. Leiter L. Monnier L. Moses A. Owens D. (2004). Postprandial glucose regulation and diabetic complications. *Arch. Intern. Med.* 164 2090–2095. 15505121 10.1001/archinte.164.19.2090

[B3] Chanoine J.-P. Mackelvie K. J. Barr S. I. Wong A. C. Meneilly G. S. Elahi D. H. (2008). GLP-1 and appetite responses to a meal in lean and overweight adolescents following exercise. *Obesity* 16 202–204. 10.1038/oby.2007.39 18223636

[B4] Chow S. Z. Speck M. Yoganathan P. Nackiewicz D. Hansen A. M. Ladefoged M. (2014). Glycoprotein 130 receptor signaling mediates alpha-cell dysfunction in a rodent model of type 2 diabetes. *Diabetes Metab. Res. Rev.* 63 2984–2995. 10.2337/db13-1121 24812426

[B5] Christensen R. H. Wedell-Neergaard A.-S. Lehrskov L. L. Legård G. E. Dorph E. B. Nymand S. (2018). The role of exercise combined with tocilizumab in visceral and epicardial adipose tissue and gastric emptying rate in abdominally obese participants: protocol for a randomised controlled trial. *Trials* 19:266. 10.1186/s13063-018-2637-0 29720225 PMC5932829

[B6] Dela F. Mikines K. J. von Linstow M. Secher N. H. Galbo H. (1992). Effect of training on insulin-mediated glucose uptake in human muscle. *Am. J. Physiol.* 263 E1134–E1143. 10.1152/ajpendo.2006.263.6.E1134 1476187

[B7] Edvardsen E. Hem E. Anderssen S. A. (2014). End criteria for reaching maximal oxygen uptake must be strict and adjusted to sex and age: a cross-sectional study. *PLoS One* 9:e85276. 10.1371/journal.pone.0085276 24454832 PMC3891752

[B8] Ellingsgaard H. Ehses J. A. Hammar E. B. Van Lommel L. Quintens R. Martens G. (2008). Interleukin-6 regulates pancreatic alpha-cell mass expansion. *Proc. Natl. Acad. Sci. U.S.A.* 105 13163–13168. 10.1073/pnas.0801059105 18719127 PMC2529061

[B9] Ellingsgaard H. Hauselmann I. Schuler B. Habib A. M. Baggio L. L. Meier D. T. (2011). Interleukin-6 enhances insulin secretion by increasing glucagon-like peptide-1 secretion from L cells and alpha cells. *Nat. Med.* 17 1481–1489. 10.1038/nm.2513 22037645 PMC4286294

[B10] Febbraio M. A. Hiscock N. Sacchetti M. Fischer C. P. Pedersen B. K. (2004). Interleukin-6 is a novel factor mediating glucose homeostasis during skeletal muscle contraction. *Diabetes Metab. Res. Rev.* 53 1643–1648. 15220185 10.2337/diabetes.53.7.1643

[B11] Fernández-Millán E. de Toro-Martín J. Lizárraga-Mollinedo E. Escrivá F. Álvarez C. (2013). Role of endogenous IL-6 in the neonatal expansion and functionality of Wistar rat pancreatic alpha cells. *Diabetologia* 56 1098–1107. 10.1007/s00125-013-2862-8 23435784

[B12] Fischer C. P. (2006). Interleukin-6 in acute exercise and training: what is the biological relevance? *Exerc. Immunol. Rev.* 12 6–33. 17201070

[B13] Heading R. C. Nimmo J. Prescott L. F. Tothill P. (1973). The dependence of paracetamol absorption on the rate of gastric emptying. *Br. J. Pharmacol.* 47 415–421. 4722050 10.1111/j.1476-5381.1973.tb08339.x PMC1776534

[B14] Holst J. J. Gribble F. Horowitz M. Rayner C. K. (2016). Roles of the gut in glucose homeostasis. *Diabetes Care* 39 884–892. 10.2337/dc16-0351 27222546

[B15] Horner K. M. Byrne N. M. Cleghorn G. J. King N. A. (2015a). Influence of habitual physical activity on gastric emptying in healthy males and relationships with body composition and energy expenditure. *Br. J. Nutr.* 114 489–496. 10.1017/S0007114515002044 26168984

[B16] Horner K. M. Schubert M. M. Desbrow B. Byrne N. M. King N. A. (2015b). Acute exercise and gastric emptying: a meta-analysis and implications for appetite control. *Sports Med.* 45 659–678. 10.1007/s40279-014-0285-4 25398225

[B17] Horner K. M. Byrne N. M. Cleghorn G. J. Näslund E. King N. A. (2011). The effects of weight loss strategies on gastric emptying and appetite control. *Obes. Rev.* 12 935–951. 10.1111/j.1467-789X.2011.00901.x 21729233

[B18] Horowitz M. Edelbroek M. A. Wishart J. M. Straathof J. W. (1993). Relationship between oral glucose tolerance and gastric emptying in normal healthy subjects. *Diabetologia* 36 857–862. 8405758 10.1007/BF00400362

[B19] Kirwan J. P. Solomon T. P. J. Wojta D. M. Staten M. A. Holloszy J. O. Jp K. (2009). Effects of 7 days of exercise training on insulin sensitivity and responsiveness in type 2 diabetes mellitus. *Am. J. Physiol. Endocrinol. Metab.* 297 E151–E156. 10.1152/ajpendo.00210.2009 19383872 PMC2711659

[B20] Kuhre R. E. Frost C. R. Svendsen B. Holst J. J. (2015). Molecular mechanisms of glucose-stimulated GLP-1 secretion from perfused rat small intestine. *Diabetes Metab. Res. Rev.* 64 370–382. 10.2337/db14-0807 25157092

[B21] Lehrskov L. L. Lyngbaek M. P. Soederlund L. Legaard G. E. Ehses J. A. Heywood S. E. (2018). Interleukin-6 delays gastric emptying in humans with direct effects on glycemic control. *Cell Metabol.* 27 1–11. 10.1016/j.cmet.2018.04.008 29731416

[B22] Martins C. Kulseng B. King N. A. Holst J. J. Blundell J. E. (2010). The effects of exercise-induced weight loss on appetite-related peptides and motivation to eat. *J. Clin. Endocrinol. Metab.* 95 1609–1616. 10.1210/jc.2009-2082 20150577

[B23] Martins C. Morgan L. M. Bloom S. R. Robertson M. D. (2007). Effects of exercise on gut peptides, energy intake and appetite. *J. Endocrinol.* 193 251–258. 17470516 10.1677/JOE-06-0030

[B24] Matsuda M. DeFronzo R. A. (1999). Insulin sensitivity indices obtained from oral glucose tolerance testing: comparison with the euglycemic insulin clamp. *Diabetes Care* 22 1462–1470. 10480510 10.2337/diacare.22.9.1462

[B25] Matthews V. B. Allen T. L. Risis S. Chan M. H. S. Henstridge D. C. Watson N. (2010). Interleukin-6-deficient mice develop hepatic inflammation and systemic insulin resistance. *Diabetologia* 53 2431–2441. 10.1007/s00125-010-1865-y 20697689

[B26] Orskov C. Rabenhøj L. Wettergren A. Kofod H. Holst J. J. (1994). Tissue and plasma concentrations of amidated and glycine-extended glucagon-like peptide I in humans. *Diabetes Metab. Res. Rev.* 43 535–539. 8138058 10.2337/diab.43.4.535

[B27] Pedersen B. K. Steensberg A. Schjerling P. (2001). Exercise and interleukin-6. *Curr. Opin. Hematol.* 8 137–141. 11303145 10.1097/00062752-200105000-00002

[B28] Phillips D. I. Clark P. M. Hales C. N. Osmond C. (1994). Understanding oral glucose tolerance: comparison of glucose or insulin measurements during the oral glucose tolerance test with specific measurements of insulin resistance and insulin secretion. *Diabet. Med.* 11 286–292. 8033528 10.1111/j.1464-5491.1994.tb00273.x

[B29] RoActemra | European Medicines Agency, (n.d.) Available at: https://www.ema.europa.eu/en/medicines/human/EPAR/roactemra. (accessed July 19, 2019).

[B30] Service F. J. Molnar G. D. Rosevear J. W. Ackerman E. Gatewood L. C. Taylor W. F. (1970). Mean amplitude of glycemic excursions, a measure of diabetic instability. *Diabetes Metab. Res. Rev.* 19 644–655. 10.2337/diab.19.9.644 5469118

[B31] Solomon T. P. J. Malin S. K. Karstoft K. Knudsen S. H. Haus J. M. Laye M. J. (2014). Determining pancreatic β-cell compensation for changing insulin sensitivity using an oral glucose tolerance test. *Am. J. Physiol. Endocrinol. Metab.* 307 E822–E829. 10.1152/ajpendo.00269.2014 25184989 PMC4216951

[B32] Song S.-N. J. Yoshizaki K. (2015). Tocilizumab for treating rheumatoid arthritis: an evaluation of pharmacokinetics/pharmacodynamics and clinical efficacy. *Expert Opin. Drug Metab. Toxicol.* 11 307–316. 10.1517/17425255.2015.992779 25491492

[B33] Suppli M. P. Lund A. Bagger J. I. Vilsbøll T. Knop F. K. (2016). Involvement of steatosis-induced glucagon resistance in hyperglucagonaemia. *Med. Hypotheses* 86 100–103. 10.1016/j.mehy.2015.10.029 26547273

[B34] Tremblay M. S. Aubert S. Barnes J. D. Saunders T. J. Carson V. Latimer-Cheung A. E. (2017). Sedentary behavior research network (SBRN) - terminology consensus project process and outcome. *Int. J. Behav. Nutr. Phys. Act.* 14:75. 10.1186/s12966-017-0525-8 28599680 PMC5466781

[B35] Treuth M. S. Schmitz K. Catellier D. J. McMurray R. G. Murray D. M. Almeida M. J. (2004). Defining accelerometer thresholds for activity intensities in adolescent girls. *Med. Sci. Sports Exerc.* 36 1259–1266. 15235335 PMC2423321

[B36] Tweedell A. Mulligan K. X. Martel J. E. Chueh F.-Y. Santomango T. McGuinness O. P. (2011). Metabolic response to endotoxin in vivo in the conscious mouse: role of interleukin-6. *Metabolism* 60 92–98. 10.1016/j.metabol.2009.12.022 20102773 PMC2889039

[B37] Wareham N. J. Phillips D. I. Byrne C. D. Hales C. N. (1995). The 30 minute insulin incremental response in an oral glucose tolerance test as a measure of insulin secretion. *Diabet. Med.* 12:931. 10.1111/j.1464-5491.1995.tb00399.x 8846687

[B38] Wedell-Neergaard A.-S. Lang Lehrskov L. Christensen R. H. Legaard G. E. Dorph E. Larsen M. K. (2018). Exercise-induced changes in visceral adipose tissue mass are regulated by IL-6 signaling: a randomized controlled trial. *Cell Metabol.* 29 1–12. 10.1016/j.cmet.2018.12.007 30595477

[B39] Wewer Albrechtsen N. J. Bak M. J. Hartmann B. Christensen L. W. Kuhre R. E. Deacon C. F. (2015). Stability of glucagon-like peptide 1 and glucagon in human plasma. *Endocr. Connect.* 4 50–57. 10.1530/EC-14-0126 25596009 PMC4317691

[B40] Wewer Albrechtsen N. J. Hartmann B. Veedfald S. Windeløv J. A. Plamboeck A. Bojsen-Møller K. N. (2014). Hyperglucagonaemia analysed by glucagon sandwich ELISA: nonspecific interference or truly elevated levels? *Diabetologia* 57 1919–1926. 10.1007/s00125-014-3283-z 24891019

[B41] Zhang X. Chen Y.-C. Terao K. (2017). Clinical pharmacology of tocilizumab for the treatment of polyarticular-course juvenile idiopathic arthritis. *Expert Rev. Clin. Pharmacol.* 10 471–482. 10.1080/17512433.2017.1300058 28293968

